# Molecular strategies to inhibit HIV-1 replication

**DOI:** 10.1186/1742-4690-2-10

**Published:** 2005-02-16

**Authors:** Morten Hjuler Nielsen, Finn Skou Pedersen, Jørgen Kjems

**Affiliations:** 1 Department of Molecular Biology, University of Aarhus, C.F. Møllers Alle, Bldg. 130, Room 404, DK-8000 Aarhus C, Denmark

## Abstract

The human immunodeficiency virus type 1 (HIV-1) is the primary cause of the acquired immunodeficiency syndrome (AIDS), which is a slow, progressive and degenerative disease of the human immune system. The pathogenesis of HIV-1 is complex and characterized by the interplay of both viral and host factors. An intense global research effort into understanding the individual steps of the viral replication cycle and the dynamics during an infection has inspired researchers in the development of a wide spectrum of antiviral strategies. Practically every stage in the viral life cycle and every viral gene product is a potential target. In addition, several strategies are targeting host proteins that play an essential role in the viral life cycle. This review summarizes the main genetic approaches taken in such antiviral strategies.

## Introduction

HIV-1 is a lentivirus belonging to the retrovirus family. The virus is diploid and contains two plus-stranded RNA copies of its genome. The approximately 9 kb RNA genome encodes at least 9 proteins, Gag, Pol, Env, Tat, Rev, Nef, Vif, Vpu and Vpr of which only the former five are essential for viral replication in vitro. HIV-1 primarily infects CD4^+ ^T-lymphocytes and monocytes/macrophages, but also astrocytes and cells of the central nervous system (brain microglial cells) are targets. The infection spreads to the lymphatic tissue that contains follicular dendritic cells that may act as a storage place for latent viruses. Over time, virus replication leads to a slow and progressive destruction of the immune system. The development of possible methods that can delay progression of the infection or block replication of HIV-1 in infected individuals has been the subject of dedicated research efforts over the past decades. One important issue is that HIV-1 makes use of the replication machinery of the host cell, which minimizes the number of potential viral targets. On the other hand, the close host-virus relationship limits the evolutionary freedom for the viral components that interact with the host molecules.

The aim of this review is to take a comprehensive look at the molecular, intracellularly based antiviral strategies that have been reported in literature, and to discuss their potential for development into clinical protocols. We will not discuss vaccine-based strategies that recently have been reviewed in [1] and [2].

### Interfering strategies against HIV-1

The inhibition strategies can be divided into two groups:

The RNA-based strategies including anti-sense RNA (or other chemically modified nucleic acids), RNA decoys (sense RNA), ribozymes, RNA aptamers, small interfering RNA (siRNA), microRNAs (miRNAs) and the protein-based strategies including transdominant negative proteins (TNPs), chimeric proteins (fusion proteins), nucleases, anti-infective cellular proteins, intracellular single-chain antibodies (sFvs) and monoclonal antibodies (Mabs). In addition, other strategies based on suicide genes, protease inhibitors and nucleoside or non-nucleoside analogues have shown to possess the ability to reduce HIV-1 replication.

**Figure 1:**
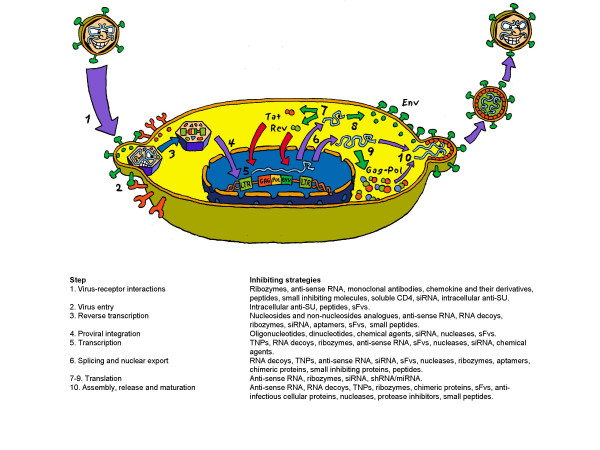
Summarization of the HIV-1 life cycle and the inhibiting strategies targeting the different steps in the viral life cycle.

The HIV-1 life cycle including the inhibiting strategies targeted against the various steps in the viral life cycle is summarized in Fig. 1 and listed in table 1. Below follows a more detailed description of the strategies taken to target individual steps of the viral life cycle. Note that strategies targeting the viral genes or mRNA directly all possess an uncertainty as to what viral function(s) are affected due to the overlapping nature of some of the reading frames [3].

**Table 1:** 

**Interfering strategy**	**Target RNA/protein**	**Interference site(s)**	**Mechanism**	**References**
Anti-sense RNA	Cellular CCR5 and CXCR4 co-receptors	Viral entry	Inhibition of CCR5 and CXCR4 gene expression	6, 18, 19
	Psi-gag and U3-5'UTR-gag-env regions	Pre-integration	Co-packaged with genomic RNA, inhibits RT in incoming virions	6
	Cellular CyPA gene	Pre-integration	The skipping of internal CyPA encoding exons reduces CyPA biosynthesis and thereby inhibits the reverse transcription	37
	Tat/TAR interaction	HIV-1 transcription	Inhibits transcriptional regulation of HIV-1 gene expression	6, 7, 45, 76
	Rev/RRE interaction	Nuclear export	Inhibits transport of unspliced and single spiced viral RNAs	6
	5'UTR	HIV-1 translation	Inhibits the translation process	6
	Psi-gag region	Viral assembly	Inhibits packaging of genomic RNA	6, 7, 45
	5'-leader-gag region	Viral assembly	Inhibits the formation of Gag and Env multimeric complexes during viral assembly.	7, 18
	Env and Vif encoding regions	Viral assembly	Inhibits env and vif gene expression	70
	Nef encoding region	Viral release	Inhibits nef gene expression and thereby CD4 and MHC I downregulation	7
	Pol encoding region	Viral maturation	Inhibits pol gene expression	70
RNA decoys	RT enzyme	Pre-integration	Competes with HIV-1 RNA for the binding of RT	6
	HIV-1 TAR region	Pre-integration	Competes with cellular tRNA_3_^Lys ^for the binding to RT and primes the reverse transcription from the TAR region instead of the PBS region	6
	Tat and Tat-containing RNA polymerase II transcription complexes	HIV-1 transcription	Inhibits Tat regulated transcription	6, 7, 18, 51
	Rev protein	Nuclear export	Recruits Rev molecules and thereby prevents their interaction with the viral transcript	6
	NC domain of the Gag protein	Viral assembly	Inhibits packaging by interfering with the NC domains ability to recognize the genomic RNA	6, 45
Ribozymes	Cellular CCR5 and CXCR4 co-receptors	Viral entry	Cleaves CCR5 and CXCR4 mRNAs	6, 18
	HIV-1 Gag and Pol encoding region and the U5 region	Pre-integration	Cleaves the viral RNA before reverse transcription is completed	6, 36
	RRE and the Rev encoding region	Nuclear export	Cleaves the viral RNA	6, 7
	U5	HIV-1 translation	Cleaves off the 5'-cap structure localized on HIV-1 mRNAs	6, 7
	Psi	Viral assembly	Cleaves HIV-1 RNAs before packaging	6, 7
	Gag encoding transcripts	Viral assembly	Inhibits the formation of multimeric Gag and Env complexes	7, 18
	SU encoding region	Viral assembly	Cleaves different conserved regions in the SU sequence	7
	Nef encoding region	Viral release	Inhibits downregulation of CD4 and MHC I	7
RNA aptamers	RT enzyme	Pre-integration	Displays high affinity and specificity for the RT enzyme and acts as templates analogues	31
	Rev protein	Nuclear export	Possesses higher affinity for Rev than the RRE sequence and can therefore interfere with Rev function	57
siRNA	Cellular CCR5 and CXCR4 co-receptors	Viral entry	Impairs the SU-chemokine co-receptor interaction	21, 22
	CD4 protein	Viral entry	CD4 protein expression inhibited	23, 24
	CD4-binding domain of the SU protein	Viral entry	Inhibits the CD4-SU interaction	26
	The viral LTR region or the vif and nef encoding regions	Pre-integration	Guides the viral genomic RNA towards a siRNA-mediated destruction	34, 52
	RT encoding region	Pre-integration	Inhibits RT gene expression	35
	Cellular CyPA gene	Pre-integration	Reduces CyPA biosynthesis and thereby the reverse transcription	37
	CA encoding region	Pre-integration	Mediates cleavage of pre-spliced viral RNA in the cytoplasm and prevents integration	23, 24, 42
	Tat encoding region	HIV-1 transcription	Inhibits Tat transactivation	35, 49, 50
	NF-κB p65 subunit	HIV-1 transcription	Inhibits NF-κB transcriptional activation	35, 49
	3'-terminus of the nef gene	HIV-1 transcription	Mediates cleavage of all spliced and unspliced RNA produced from the provirus	42
	Rev transcript	Nuclear export	Inhibits Rev mediated export of unspliced and single spliced RNAs	49, 61
	Gag and Nef encoding regions	HIV-1 translation	Mediates cleavage of both spliced and unspliced RNA produced from the provirus	23, 24, 34, 42
shRNA/ miRNA	Nef encoding region	HIV-1 translation	nef shRNAs act by blocking RNA stability or RNA translation	62
Transdominant negative proteins (TNPs)	Interactions between Tat/TAR complex and cellular co-factors	HIV-1 transcription	Tat-mutants inhibit the function of the Tat protein by recruiting important cellular co-factors	7, 18, 45
	Rev protein	Nuclear export	Rev-mutants e.g. act by preventing the interaction with cellular co-factors or by sequestering the Rev protein in the cytoplasm	7, 18, 25, 57, 58, 59
	Cellular Sam68	Nuclear export	Sam68 mutants inhibit Sam68 transactivation of RRE and Rev function	60
	Cellular Tsg101	Viral assembly	Tsg101 mutants inhibit the transport of the Gag polyprotein into multivesicular bodies	71
	Vif protein	Viral assembly	Vif mutants block an early processing of the Gag protein	66
	Cellular INI1	Viral assembly	INI1 mutants e.g. interact with the integrase domain of the Gag-Pol polyprotein and interfere with prober multimerization of Gag and Gag-Pol	39
	The formation of Gag and Env multimeric complexes	Viral assembly	E.g. interferes with complex formation	4, 6, 18
	Nef protein	Viral release	Nef mutants e.g. inhibit CD4 downregulation	66
	SU protein	Viral release	Overexpressed CD4 variants bind and sequester virion progeny within the cell	19
	HIV-1 protease	Viral maturation	Pro-mutants prevent protease activation	7
Chimeric / fusion proteins	SU protein	Viral entry	A tetrameric version of sCD4, PRO542, which is fused to the conserved region of IgG2, prevents the CD4-SU interaction	8, 13
	Proviral DNA	Pre-integration	An IN targeted sFv-nuclease fusion protein associates with the pre-integration complex and cleaves proviral DNA after integration has occurred	7, 18
	TAR element	HIV-1 transcription	Designed Tat-nuclease fusion proteins recognize and cleave all HIV-1 RNA transcripts	5
	RRE sequence	Nuclear export	Designed Rev-nuclease fusion proteins recognize and cleave all HIV-1 RNAs carrying the RRE sequence	5
	Rev protein	Nuclear export	A NS1RM-Rev mutant, with a dominant retention activity, forms mixed oligomers together with Rev and inhibits nuclear export	7, 57
	The TAR and RRE elements	HIV-1 transcription / nuclear export	A designed fusion protein, Tev, containing the RNA binding domains of both Tat and Rev fused to a nuclease, inhibits both early and late viral gene products	5
	Viral genomic RNAs	Viral assembly	Gag-, Vpr- and Nef-nuclease fusion proteins cleaves viral RNA, either during or after the viral assembly	5, 7
	Psi-element	Viral assembly	A NC-nuclease fusion protein recognizes and cleaves all unspliced RNAs in the cytoplasm	5
	HIV-1 protease	Viral maturation	An overexpressed Vpr fused to several protease cleavage sites overwhelms the protease activity by a competitive mechanism	7, 74
Nucleases	Tat encoding region	HIV-1 transcription	Inhibits Tat transactivation	6, 7, 45
	TAR element	HIV-1 transcription	Inhibits Tat transactivation	6, 7, 45
Chemokine ligands	Cellular CCR5 and CXCR4 co-receptors	Viral entry	E.g. interacts directly with the co-receptors, mediates receptor blockade or mediates receptor down-regulation	8, 9, 11, 12, 13, 14, 16
Anti-infectious cellular proteins	SU protein	Viral entry	A truncated form of CD4, sCD4, inhibits the fusion event by binding to the SU protein and thereby extending the distance to the TM protein	8, 13, 19
Intracellular antibodies (sFvs)	SU protein	Viral entry	Inhibits the CD4-SU interaction	18
	The TM pre-hairpin intermediate	Viral entry	Inhibits the interaction between the fusion peptide and the cell membrane	29
	RT enzyme	Pre-integration	Inhibits RT function	7, 18
	IN enzyme	Pre-integration	Inhibits IN function	7, 18
	Tat protein	HIV-1 transcription	Interacts with the Tat protein and restrains it in the cytoplasm	7, 18
	Rev protein	Nuclear export	Recruits Rev in the cytoplasm	7, 18, 25, 57
	The CD4 binding region of the SU protein	Viral assembly	Interacts with the Env protein and restrains it in the ER	7, 18
Monoclonal antibodies (Mabs)	Cellular CCR5 and CXCR4 co-receptors	Viral entry	E.g. inhibit the SU-chemokine co-receptor interaction, HIV-1 fusion or entry	12
	Extracellular loop on CCR5	SU-chemokine co-receptor interaction	Inhibits HIV-1 fusion and entry	12
Nucleoside analogues (NRTIs)	RT enzyme	Pre-integration	Prevents the continued polymerization of the DNA chain	8
Non-nucleoside analogues (NNRTIs)	RT enzyme	Pre-integration	Interact directly and non-competitively with the RT enzyme and inhibits its function	8
Integrase inhibitors (Oligonucleotides, dinucleotides and chemical agents)	IN enzyme	Pre-integration	These inhibiting agents either block the catalytic function of the IN enzyme by binding to the integrase binding site located in the viral DNA, or by interacting with the catalytic core domain of the IN enzyme itself	40, 41
Protease inhibitors	Protease enzyme	Viral maturation	Act as transition state analogous and bind to the protease more tightly than the natural substrate	11, 8, 73
Examples of other inhibiting agents	Cellular CCR5 and CXCR4 co-receptor	Viral entry	Chemokine ligands potently inhibit the SU-chemokine co-receptor interaction	8, 9, 10, 11, 12, 13
	Cellular CCR5 and CXCR4 co-receptors	Viral entry	Designed peptides e.g. act by disrupting helix-helix interactions, which may influence co-receptor structure, or by associating with the co-receptor surfaces and thereby inhibit the interaction with the SU protein	8, 12
	Cellular CXCR4 co-receptor	Viral entry	AMD3100, a small organic molecule, acts by spanning the main ligand-binding cavity of CXCR4, which constrains the co-receptor in an inactive conformation	12
	Cellular CCR5 co-receptor	Viral entry	Cyclophilin-18, a protein derived from T. Gondii acts as a CCR5 antagonist and thereby inhibits fusion and infectivity of R5 HIV-1 isolates	17
	SU protein	Viral entry	CV-N, a 11 kDa protein with high affinity for the SU protein, inhibits the SU-CD4 interaction	15
	The N- and C-peptide regions on the TM pre-hairpin intermediate	Viral entry	Designed N-, C-, and D-peptides interacts with the pre-hairpin intermediate and inhibit the fusion event	13, 27, 28
	RT enzyme	Pre-integration	Small peptides, about 15–19 amino acid long, act by interfering the dimerization process of the RT enzyme	30
	The Tat/TAR interaction	HIV-1 transcription	The TR87 compound acts by competing with Tat for binding to TAR-RNA	46
	Protein /TAR RNA interaction	HIV-1 transcription	Pyrrolo [2,1-c][1,4]benzodiazepine-oligopyrrolo hybrids act by interrupting binding of cellular proteins and Tat to the TAR-RNA	47
	Protein /TAR RNA interaction	HIV-1 transcription	Aromatic polyamidines carrying a Br atom inhibit cellular and viral protein-TAR RNA interactions	48
	Cellular NF-κB	HIV-1 transcription	NF-κB activity is inhibited by minocycline, a second-generation tetracycline	38, 54
	Rev	Nuclear export	Peptides targeted against the NES domain inhibit Rev function	57
	The cellular protease furin	Viral assembly	Peptides mimicking a conserved target sequence inhibit furin activity and thereby cleavage of the Env protein within the ER	72
	HIV-1 infected cells	All	A Tat-Casp3 fusion protein induces apoptosis after cleavage and activation by the HIV-1 protease	79

### Virus-receptor interaction and entry

HIV-1 infection is initiated by binding of the virion gp120 surface subunit (SU protein) to the CD4 receptor. The SU protein is attached to the virus by a non-covalent binding to the gp41 transmembrane subunit (TM protein). Both SU and TM are proteolytically cleaved from the Envelope (Env) precursor protein by a cellular convertase, furin, within the endoplasmatic reticulum (ER). Both remain noncovalently attached and are targeted to the host plasma membrane by vesicular transport. The SU protein is responsible for receptor recognition on CD4^+ ^T-lymphocytes and the TM protein mediates the fusion between the viral membrane and the host cell membrane [4,5].

Binding to CD4 induces a structural alteration in SU that exposes the binding site for a co-receptor of the chemokine family. The major co-receptors required for entry of HIV-1 are the chemokine receptor molecules CCR-5 (R5 HIV-1 isolates) and CXCR-4 (X4 HIV-1 isolates), which are used by monocytes/macrophage-tropic and T-cell tropic HIV-1 viruses, respectively [6]. When the SU protein binds to the co-receptor the result is another structural alteration exposing the N-terminal part of TM. This part, also known as the fusion-peptide, mediates the fusion between the viral and host membranes. The Env protein is also capable of mediating fusion between infected and non-infected cells by a process known as syncytium formation [4,7,8].

Current strategies are targeting particularly the CD4-SU interaction, the SU-chemokine co-receptor interaction, and the TM-mediated virus-cell membrane fusion process.

#### The SU-chemokine co-receptor interaction

CCR-5 and CXCR-4 co-receptors have specific chemokine ligands/antagonists that possess the ability to block the virus infection. The molecules that bind to the co-receptors can be divided into four categories: naturally occurring chemokines and their derivatives, peptides and small molecules (< 1 kDa), and Mabs, which recognize epitopes on for instance the extracellular domains of certain receptors.

Examples of chemokine ligands (beta-chemokines) that inhibit infection of R5 isolates include RANTES, a physiological ligand for the HIV-1 co-receptors CCR3 and CCR5. RANTES is actively secreted by normal T-cells. Derivatives of this peptide have been used, including aminooxypentane (AOP)-RANTES [9], and from a recent study, N^α^-(n-nonanoyl)-*des-*Ser^1 ^[L-thioproline^2^, L-α-cyclohexyl-glycine^3^] RANTES (PSC-RANTES) [10]. RANTES is an antagonist that besides having the ability to interact with CCR5 also has a downregulating effect on the co-receptor. RANTES can however induce chemotaxis and promote unwanted inflammatory side effects. Therefore AOP-RANTES was created by chemical modification of the amino terminus. This analogue does not promote any inflammatory side effects, and in addition it can prevent chemotaxis induced by e.g. RANTES. AOP-RANTES is a very strong antagonist that has a high affinity for CCR5, elicits rapid endocytosis of CCR5, and prevents recycling of the co-receptor back to the surface. PSC-RANTES is chemically identical to native RANTES except for the substitution of a nonanoyl group, thioproline, and cyclohexylglycine for the first three N-terminal amino acids of the native protein. This analogue acts in the same way, but has shown more potent *in vitro *antiviral activity than AOP-RANTES. Furthermore, it has successfully protected rhesus macaques from intravaginal exposure to a chimeric simian/human immunodeficiency virus containing an R5-tropic envelope of HIV-1 [10].

In addition to RANTES and its derivatives, the chemokine ligands macrophage inflammatory proteins 1alpha/beta (MIP-1alpha and MIP-1beta) also show an inhibiting effect by mediating a receptor blockade [8,11-13]. Examples of chemokine ligands that inhibit infection of X4 isolates include stromal cell-derived factor-1alpha (SDF-1alpha) and its derivatives that inhibit HIV-1 fusion and entry by minimizing the accessibility to the co-receptor on the cell surface and by inhibiting the SU-CXCR4 interaction [9,11-13].

The CCR5 amino-terminal domain is thought to play an important role in virus fusion and entry. This knowledge has been utilized in the development of anti-CCR5 Mabs whose epitopes include residues in the amino-terminal domain. Mabs of this kind strongly inhibit SU binding to CCR5 but only moderately inhibit HIV-1 fusion and entry [12]. Another type of Mab, the anti-ECL2 Mab whose epitopes include residues from one of the extracellular loops on CCR5 (ECL2), potently inhibits HIV-1 fusion and entry, but only moderately inhibits SU binding [12]. PRO 140, also an anti-CCR5 Mab, inhibits viral fusion with the cell membrane at concentrations that do not prevent the CCR5 chemokine receptor activity. It binds a complex epitope spanning multiple extracellular domains on CCR5, and although it acts as a weak antagonist it does not induce signaling or downregulation of CCR5. It is thought that the antiviral effect is exerted through a mechanism involving receptor blockade [14]. Mab 12G5 is a monoclonal antibody that recognizes an epitope on CXCR4. This epitope is also present in ECL2, and binding inhibits HIV-1 fusion [12,15]. A potential disadvantage of this strategy is that binding of the antibody to a receptor may trigger unwanted signal transduction [14,16].

Peptides, resembling the CCR5 transmembrane helices, inhibit HIV-1 replication and chemokine signaling by disrupting helix-helix interactions, which may influence the CCR5 structure [12]. T22 is a positively charged cyclic 18-mer antimicrobial peptide, which presumably inhibits SU-CXCR4 interaction by associating with the negatively charged surface of CXCR4 [8,12]. A truncated form of SDF-1alpha, consisting of the 16 amino-terminal residues of SDF-1alpha, also seems to possess such a blocking effect [12].

Recently, a new kind of CCR5 antagonist has been discovered in a protozoan parasite, *Toxoplasma gondii *[17]. This protein, cyclophilin-18 (C-18), has several potential antiviral properties including CCR5 binding, induction of the production of interleukin-12 (IL-12) from murine dendritic cells, inhibition of fusion and infectivity of R5 isolates by co-receptor antagonism and blocking of syncytia formation.

Small organic molecules, such as AMD3100, potently inhibit HIV-1 replication by an interaction with residues present on one of the CXCR4 extracellular loops, ECL2, and residues within a transmembrane helix, TM4. Upon binding to these residues AMD3100 spans the main ligand-binding cavity of CXCR4, which probably constrains the co-receptor in an inactive conformation [12].

Individuals with a homozygous deletion in the gene encoding CCR5 are healthy and protected against HIV-1 transmission, which suggests that down regulation may not pose any clinical side effects. This knowledge has led to the development of strategies that directly target the mRNA encoding CCR5 or CXC4, either by ribozymes [6,18], anti-sense RNA [6,18,19] or RNAi [20]. The latter strategy, the siRNA approach, has led to successful blocking of HIV-1 entry, protection of cells from infection and delay of virus replication [21-24]. Interestingly, it is thought that single-stranded siRNAs (the anti-sense strand of a siRNA duplex) act through the same RNAi pathway, but at a later stage than double-stranded siRNA, thereby requiring less time to exert their antiviral activity [21,25].

#### The CD4-SU interaction

Soluble CD4 (sCD4) is an anti-HIV-1 protein, which can be expressed and secreted from genetically engineered cells. It is a truncated form of the CD4 receptor, composed of the ectodomain that inhibits laboratory-adapted strains of HIV-1. sCD4 probably prevents the binding of the virus to the cell, by binding directly to Env, or indirectly by inducing or repressing cellular factors that influence the viral gene expression [18,19].

When sCD4 binds to SU it acts by extending the distance to TM, which inhibits the fusion. But when used against primary isolates, sCD4 was much less successful because of a lower affinity for sCD4. Surprisingly, some isolates became more infectious upon sCD4 treatment. An explanation for this may be that an interaction between the SU protein and sCD4 induces changes in SU, allowing it to bind the co-receptor with higher affinity or increased kinetics. In addition this interaction can eventually facilitate the fusion of HIV-1 with CD4^- ^cells expressing the co-receptor [13]. This has led to the development of a tetrameric version of sCD4, PRO542, in which the SU-binding region of CD4 is fused to the conserved region of human immunoglobulin IgG2. This fusion protein has a high affinity for the SU protein and has shown promising results in phase I clinical trials [8,13].

siRNA-directed silencing of CD4 mRNA expression has been shown to specifically inhibit HIV-1 entry and thus HIV-1 replication [23,24]. However, CD4 silencing in vivo may interfere with its role in normal immune function. Thus an approach targeting the CD4-binding domain of the SU protein would be more relevant. This has successfully been achieved by expressing a 0.5 kb dsRNA containing the major CD4-binding domain of the SU protein, as the target of the env gene. By this approach it has been possible to significantly suppress the expression of the HIV-1 CA-p24 antigen in human peripheral blood mononuclear cells (PBMCs) and in HeLa-CD4^+ ^for a relatively long period of time [26].

Strategies based on the intracellular expression of antibodies specific for the HIV-1 envelope (anti-SU) have also been shown to inhibit virus replication. This strategy is based on the usage of sFvs, containing the smallest structural domain that still possesses complete antigen and binding-site specificity of the parental antibody. They are secreted into the medium where they probably act as inhibitors by direct interaction with the viral proteins [18] to neutralize the virus [19].

Cyanovirin (CV-N), an 11 kDa protein originally isolated from cyanobacteria, potently inactivates diverse strains of HIV-1. It has a high affinity for the SU protein, and when bound it inhibits the SU-CD4 interaction. CV-N possesses the advantage that even high concentrations are non-toxic and it is an extremely stable protein. CV-N has also been coupled to a cytotoxin (Pseudomonas exotoxin), thereby selectively killing HIV-1 infected SU-expressing cells [15].

#### The TM-mediated virus-cell membrane fusion

As the SU protein binds to CD4, it initiates conformational changes in SU, making the interaction between the SU protein and the co-receptors more favorable. After attachment to the co-receptor further conformational changes occur in both the SU and TM proteins, thus weakening their interaction. During this process a transitory pre-hairpin intermediate of the TM protein is created, freeing the previously buried fusion peptide to interact with the host-cell membrane. This exposes the N-peptide and the C-peptide regions on the pre-hairpin intermediate that have been targets for several inhibiting strategies including synthetic C-peptides, N-peptides and sFvs.

C-peptides are based on the C terminal end of the fusion peptide, and mimics this part of the fusion peptide when it has its correct fusogenic conformation. T-20, a 36-amino acid C-peptide, is a potent inhibitor of HIV-1 infection. It acts through a dominant negative mechanism and interacts by binding to a conserved domain on the N-peptide present in the pre-hairpin intermediate. The function of this domain is to mediate a structural change, which allows the pre-hairpin intermediate to form a fusogenic hairpin state. Binding of T-20 inhibits this process and thereby impedes fusion. Disadvantages of the C-peptide strategy are the cost of C-peptide synthesis and the relatively large amounts necessary for an antiviral effect. In addition, their size makes them non-amenable to oral routes of entry and they must be injected instead [13,27,28].

The 5-Helix is a 25 amino acid N-peptide consisting of five of the six helixes constituting the C-peptide. The peptide is presumed to inhibit fusion, through binding with high affinity to the C-peptide. However because N-peptides have a strong tendency to aggregate the inhibition could also be due to their intercalation into the TM amino-terminal coiled coil [27,28].

A third kind of peptides named D-peptides have also proven effective. These peptides are small 16–18 D-amino acids residues that specifically bind to three hydrophobic pockets present at the end of the N-peptide. Since such peptides are unnatural, they are resistant to proteolytic degradation, which makes them attractive for clinical use [13,27].

Recently, a non-neutralizing antibody directed against epitopes exposed on the fusion peptide has been reported to possess antiviral effect [29]. This antibody does not neutralize HIV-1 entry when produced as a soluble protein. However, when expressed on the cell surface as a membrane-bound sFv, it is turned into a neutralizing antibody, which markedly inhibits HIV-1 replication and cell-cell fusion by a mechanism that is thought to involve an interaction with the exposed fusion peptide. This results in inhibition of the subsequent fusion process. In the same study, this sFv was targeted into the ER and trans-Golgi network of HIV-1 susceptible cell lines where it was found to significantly block the maturation process of the viral Env protein resulting in an impairment of viral assembly.

### Reverse transcription and proviral integration

After fusion the viral core enters the cytoplasm and the viral RNA is copied into double-stranded cDNA. This process is mediated by the viral reverse transcriptase (RT) enzyme in a complex consisting of RT, the viral genome, and a cellular tRNA_3_^lys^. The latter acts as primer and initiates negative strand DNA synthesis by binding to the primer binding site (PBS) region, located immediately 3' to the U5 region [6,4]. RT possesses three essential activities important for replication of the virus: RNA-dependent DNA polymerase (i.e. reverse transcriptase), RNase H activity (i.e. cleaves the genomic RNA in RNA/DNA hybrids during DNA synthesis), DNA-dependent DNA polymerase activity (i.e. for synthesis of the second strand of the proviral DNA) [6,4].

Because RT is essential for viral replication it has been one of the most popular targets. This has led to the following antiviral strategies.

#### RT-targeted strategies

Inhibiting strategies against RT involve the utilization of nucleosides and non-nucleosides. The nucleoside analogues lack the 3'-hydroxyl group, prevent the continued polymerization of the DNA chain, and are usually named nucleoside reverse transcriptase inhibitors (NRTIs). Clinically approved examples include Zidovudine (AZT), Didanosine (ddI), Zalcitabine (ddC), Lamivudine (3TC), Abacavir succinate and Stavudine (d4T) [8].

The non-nucleoside analogues, often referred to as non-nucleoside reverse transcriptase inhibitors (NNRTIs), act at the same step in the viral life cycle as the nucleoside analogous, but by a significantly different mechanism. Instead of acting as false nucleosides, the NNRTIs bind directly and non-competitively to RT in a way that inhibits the enzyme's activity. Examples of clinically approved NNRTIs include Nevirapine, Delaviridine and Efavirenz [8].

NRTIs bind to the deoxynucleoside triphosphate-binding pocket, which is formed partly by the template-primer nucleic acid and partly by the protein surfaces. NNRTIs bind to a hydrophobic pocket exclusively present in the RT enzyme of M subtype HIV-1. When used in combination they have a more pronounced antiviral effect.

The RNA decoy strategy aimed at RT involves the expression of RNAs lacking the PBS region, thus preventing it from acting as template for reverse transcription. The RNA competes with HIV-1 RNA when RT makes the first jump during the first strand transfer [6]. Another decoy was designed to be co-packaged together with genomic RNA into new virions where it competes subsequently with genomic RNA for RT binding [6]. Also, a designed tRNA_3_^Lys ^mutant containing an 11 nucleotide 3'-end complementary to the HIV-1 TAR region, shows an inhibiting effect. This mutant competes with cellular tRNA_3_^Lys ^for the binding to RT and primes reverse transcription from the TAR region instead of the PBS region [6].

Other strategies against the RT enzyme involve the usage of small peptides, about 15–19 amino acids long, that inhibit RT activity by interfering with the dimerization process of the RT enzyme. The amino acid sequence corresponds to the so-called connection domain of RT, in particular a tryptophan-rich 19-mer sequence corresponding to residues 389–407, which efficiently inhibits viral replication [30]. Likewise, strategies based on intracellular expression of sFvs [7,18] and RNA aptamers [31-33] targeted against the RT enzyme are potent inhibitors of HIV-1 replication. The aptamers all recognize the same template-primer-binding cleft on RT. Some of these RNA aptamers have the potential to form pseudoknot-like secondary structures, which mimic the conformation of the template-primer when associated with the RT enzyme. Thus, these aptamers are termed template analogue RT inhibitors (TRTIs). Selectivity of the RNA aptamers is directly related to their three-dimensional structure. Utilization of the TRTI aptamers has the following benefits: 1) Aptamers have a unique specificity and a strong binding affinity for the RT enzyme. 2) Aptamers inhibit the RT enzyme competitively and will unlikely inhibit other viral or cellular proteins, thus minimizing the risk for any appreciable toxic side effects. 3) Since aptamers are expressed in the infected cell, the aptamers will be co-packaged into new virions and inhibit the next round of replication. 4) Because of the large interface of the aptamer-binding pocket, the risk of escape mutants is significantly reduced. Furthermore, mutations in essential binding domains, such as the template-primer-binding pocket, will likely impair the binding of the RT enzyme to the viral genome [31].

Anti-sense RNAs designed to be complementary to the Psi-gag and the U3-5'UTR-gag-env regions have been shown to inhibit RT in new virion particles. They are co-packaged together with the genomic RNA into the virus progeny, and inhibit reverse transcription by hybridizing to the genomic RNA [6].

siRNAs directed against several regions of the HIV-1 genome, including the viral long terminal repeat (LTR) and the accessory genes vif and nef have provided evidence that the viral genomic RNA, as it exists within the virion as a nucleoprotein reverse transcription complex, is amenable to siRNA-mediated degradation [34]. In addition, siRNAs targeted against the RT gene alone have shown potent inhibition of HIV-1 replication in MAGI cells [35].

Hammerhead ribozymes targeted against the HIV-1 gag region will cleave the viral RNA before reverse transcription is completed [6]. Hairpin ribozymes, designed to cleave a conserved site in the U5 region of the HIV-1 RNA can likewise inhibit replication [6]. Especially the tRNA^Val^-U5-ribozyme has shown promising results and is currently being tested in clinical trials. Moreover, hairpin and hammerhead ribozymes targeted against the HIV-1 pol region also show promising results [6,36]. In the latter strategy a hammerhead ribozyme has successfully been packaged into virions by linking it to the portion of the HIV-1 genome that provides the packaging sequence [36]. This intravirion targeting ribozyme has in the same study shown higher virus-suppressing activity than a nonpackageable counterpart.

Since host tRNA_3_^Lys ^is being packaged into new virus particles, this molecule is often used when ribozymes have to be co-packaged. An example is the tRNA_3_^Lys^-hammerhead ribozymes targeted against the PBS region. Besides cleaving the HIV-1 RNA, the tRNA_3_^Lys^-ribozyme inhibits reverse transcription by competing with host tRNA_3_^Lys ^for RT binding and/or for the binding to the PBS sequence. Also, when bound to the PBS, the tRNA_3_^Lys^-ribozyme is unable to prime reverse transcription [6].

In a study closely related to the earlier mentioned CCR5 antagonist, C-18, human cyclophilin A (CyPA) has been shown to be incorporated into HIV-1 during virion assembly through interaction with an exposed proline-rich loop within the capsid domain of Gag [37,38]. CyPA is required for efficient viral replication but not for cell viability meaning that its cellular function is probably being compensated for by other factors. It has been proposed that CyPA enhances HIV-1 infectivity during early post-entry events, but may also be required for viral entry. The proposed molecular interaction that underlies this enhancement is the CyPA proteins ability to mask the binding site for the human host restriction factor Ref1 and thereby counteracting its inhibitory activity, allowing reverse transcription to be completed. In an attempt to reduce CyPA biosynthesis, two different anti-sense strategies were used [37]. In one approach internal CyPA exons are skipped by means of modified derivatives of U7 small nuclear RNA (snRNA). U7 snRNA is the RNA component of the U7 small nuclear ribonucleoprotein (snRNP) involved in histone RNA 3'-end processing. By inserting appropriate anti-sense sequences into U7 snRNA it has successfully been converted from a mediator of histone RNA processing to an effector of alternative splicing. The other strategy involves the use of hairpin siRNA constructs targeting two different parts of the CyPA coding region. Both strategies greatly reduced the levels of CyPA, creating CEM-SS T-cells that sustain HIV-1 replication.

The next step is the translocation of the cDNA containing capsid into the nucleus. This process is mediated by independent pathways involving either the Vpr accessory protein, the matrix protein (MA) or the integrase (IN) protein. Vpr is thought to mediate the nuclear import of the preintegration complex through the nuclear pore complex (NPC) in non-dividing cells by interacting directly with proteins in the NPC. This transfer of viral DNA is mediated by a nuclear localization signal present in the Vpr protein. Furthermore, it has been shown that Vpr is involved in arresting HIV-infected cells in the G2 phase of the cell cycle, where the virus production has been shown to reach a maximum level [4-7]. Integration of proviral DNA is mediated by the viral IN enzyme by a process that requires the host protein integrase interactor 1 (INI1 / hSNF5). IN consists of an N-terminal zinc finger domain, a catalytic core domain, and a C-terminal domain that is important for binding HIV-1 LTR DNA [39,40]. It has two enzymatic functions; DNA cleavage and insertion of the provirus into the genome of the host [4,7]. IN recognizes short inverted repeats (att sites) at both ends of the proviral DNA and cleaves an AT overhang at the 5' end. Then it catalyzes the non-specific cleavage of the host genome and the subsequently ligation of the 5' overhang to the cellular genome [4].

Several strategies aiming at the IN function have been reported:

#### IN-targeted strategies

IN has no known functional analogue in human cells and is therefore an appealing target for inhibiting strategies, which generally involves the usage of oligonucleotides, dinucleotides and different kinds of chemical agents, such as dicaffeoylquinic acids (DCQAs) [40] and 2,4-dioxobutanoic acid analogous [41]. The integrase binding site in the U3 LTR region of the viral DNA contains a purine motif, 5'-GGAAGGG-3'. This motif has selectively been targeted by oligonucleotide-intercalator conjugates that interact with the viral DNA through triplex formation, thus blocking the catalytic functions of the IN enzyme [41]. Disadvantages of these compounds include the low intracellular permeability and the high mutation rate of HIV-1 that may result in nucleotide substitutions in the LTR.

The inhibiting effect of a dinucleotide, named pdCpIsodU, is due to its ability to interact with the catalytic core domain [41]. This molecule consists of a natural D-deoxynucleoside and an isomeric L-related deoxynucleoside joined together through a stereochemically unusual internucleotide phosphate bond, which makes the molecule resistant to 5'- and 3'-exonucleases. Through binding the molecule inhibits both the 3'-processing and the DNA strand transfer step.

DCQAs are non-competitive inhibitors that act by irreversible binding to the catalytic core domain. The exact chemical mechanism for this anti-IN activity is unknown, but it is thought to be caused by a simple redox-process. Two examples are 1-Methoxy-3,5-dicaffeoylquinic acid and 3,4-Dicaffeoylquinic acid. Both are relative non-toxic [40]. Finally, 2,4-dioxobutanoic acid analogous have been reported to possess potent anti-IN activity through inhibition of the DNA strand transfer step [41].

sFvs interacting with different domains on IN have been isolated, and by fusion with a nuclease, a fusion protein is created that can interact with IN in the pre-integration complex, leading to cleavage of proviral DNA. Likewise IN-specific sFvs have been shown to be inhibitory to HIV-1 replication [7,18].

Finally, siRNAs targeted against the capsid protein, p24-siRNA, is thought to interact with the gag gene in the unspliced viral RNA when present in the cytoplasm. Thereby, the viral RNA genome is cleaved before integration occurs [23,24,42].

### HIV-1 transcription

Transcriptional regulation of HIV-1 gene expression is controlled by co-operative and cell-specific interactions between several host cells transcription factors, including AP-1, NF-κB, NF-AT, NF-IL-6, CREB, IRF, Sp1, LEF-1/TCF-1α, Ets-1 and USF, and the viral Tat protein [5,7,43]. The Tat protein recognizes a stem-loop structure, the trans-activation responsive element region (TAR), located in the 5'-end of the primary transcript (R region). Tat recruits a cellular co-factor, positive transcription elongation factor b (P-TEFb), composed of human cyclin T1 (hCycT1) and CDK9 (a CTD kinase). The hCycT1 component binds to the activation domain of Tat thereby increasing the affinity for TAR. This results in the formation of a Tat/TAR complex. Next, CDK9 phosphorylates the carboxy-terminal domain of the host cell RNA polymerase II, which stimulates the elongation process and thereby the overall transcriptional efficiency [4,44].

The Tat/TAR interaction is essential for activation of HIV-1 transcription and is therefore a popular target for inhibiting strategies. Another reason for choosing strategies directed against this step is that the Tat-TAR interaction is highly conserved. Thus the chance for development of escape mutants is very low, due to the fact that mutations in either Tat or TAR will cause an impaired interaction between them and thereby abolish HIV-1 replication.

One strategy is to express a Tat protein that displays a transdominant negative phenotype, which can inhibit the replication of HIV-1. These proteins act as competitors for Tat binding to an essential substrate or co-factor, or alternatively by associating with wild-type monomers to form an inactive mixed multimer. Examples include Tat proteins containing mutations in the activating domain, the protein-binding domain, or in the TAR binding domain [7,18,45]. An obvious disadvantage of this strategy is, as mentioned earlier, the mutants' ability to recruit co-factors important for maintaining of a normal cellular function. Tat function can also be impaired by using a single-chain antibody, sFv-Tat. When sFv-Tat interacts with the Tat protein, it restrains Tat in the cytoplasm, thus hindering its transcription-regulating function in the nucleus [7,18].

Several studies have shown promising results in blocking the interaction of cellular TAR RNA-binding proteins and viral Tat protein to TAR RNA. For instance these include a study in which a compound termed TR87 directly competes with Tat for binding to TAR [46], and a study involving pyrrolo [2,1-c][1,4] benzodiazepine-oligopyrrolo hybrids, which appear to interrupt protein/TAR RNA interactions and Tat-induced LTR-driven HIV-1 transcription [47], and finally a study were two aromatic polyamidines carrying a halogen atom, termed TAPB-Br and TAPP-Br, have demonstrated the potential to inhibit cellular and viral protein-TAR RNA interactions [48].

An inhibiting effect has also been observed using anti-sense-, nuclease-, or siRNA-based strategies directed against tat mRNA [6,7,35,45,49]. Notably, a recent study has demonstrated that tat siRNA delivered as pre-miRNA precursor is 80 % more effective than tat siRNA expressed as conventional short hairpin RNAs (shRNAs) [50]. Finally, anti-sense RNAs and nucleases targeting the TAR element have also shown promise as antivirals [5-7,45].

A Tat-nuclease fusion protein has been engineered by fusing the HIV-1 TAR RNA binding domain of HIV-1 Tat with the RNase H domain of HIV-1 RT. Since TAR is present at the 5' and 3' ends of all HIV RNAs, this Tat-nuclease can recognize and cleave all HIV-1 RNA transcripts. The Tat protein cycles in and out of the nucleus and the cleavage of HIV-1 transcripts should therefore take place both in the nucleus and in the cytoplasm [5].

TAR decoys represent another example of a suitable strategy. These sense RNAs act by interacting with Tat-containing RNA polymerase II transcription complexes that assemble on the HIV-1 promoter [7]. In addition the TAR decoy RNAs solely recruit the Tat protein and the cellular co-factor, P-TEFb, which is necessary for Tat-mediated transactivation [18]. To make this strategy more effective, the development of polymeric TAR decoys has been accomplished. Constructs with up to 50 TAR elements have been reported, but unfortunately these constructs also recruit essential functional cellular co-factors [6,18]. A TAR decoy based on the element of HIV-2 (TAR-2) has been shown to suppress HIV-1 replication more effective that the decoy based on HIV-1. The explanation for this is that the TAR-2 structure possesses three separate loop regions and may therefore more effectively compete with the single stem-loop structured TAR in HIV-1 for loop-binding cellular factors [51].

Besides targeting the Tat encoding regions, siRNAs have been directed against the Gag [23,24,42] and Nef [23,34] encoding regions. Both siRNAs show antiviral effects. The gag-targeted siRNA (p24-siRNA) is identical to the p24-siRNA utilized when inhibiting the pre-integration step and acts in the same manner. Since the nef gene is located in the 3' end of the HIV-1 genome and in many of the viral transcripts, a siRNA directed against the Nef encoding region will reduce the number of viral transcripts. Also a 3'-LTR directed siRNA has shown potently to suppress viral replication [42]. The 3'-LTR region was chosen, as it is in the noncoding sequence before the poly(A) tail, except for the Nef encoding RNA. Thus, by this specific siRNA approach it is possible to target both spliced and unspliced RNA produced from the provirus, whereas the p24-siRNA approach only targets the unspliced viral RNA. By combining these siRNAs a synergistic effect has been observed [23,24,34,42]. The high specificity of the RNAi approach also makes it vulnerable to inactivating mutations in the viral genome as was observed in a recent study [52]. Another promising strategy involving siRNAs includes targeting of the NF-κB p65 subunit [35]. The NF-κB p65 subunit is a key component for NF-κB transcriptional activation of HIV-1. During the early phases of HIV-1 infection in activated T lymphocytes, NF-κB binding to the HIV-1 LTR serves to stimulate the generation of at least some full-length transcripts for synthesis of Tat, which then stimulates the transcriptional elongation process. This is supported by the observation that siRNAs targeted against the NF-κB p65 subunit show a decrease of HIV-1 replication in MAGI and Jurkat cells. However, the NF-κB proteins are also critical for the regulation of immune function. They regulate the expression of a variety of genes encoding cytokines and cytokine receptors, chemokines, cell adhesion molecules, and cell surface receptors that are critical for T- and B-cell function. Therefore further studies are required before p65-siRNAs can be used in clinical trials [3,35,49]. Finally, a siRNA mediated knockdown of cellular P-TEFb has surprisingly shown to decrease HIV-1 transcription and viral replication without being lethal to the cell. It seems that there is a critical threshold of P-TEFb kinase activity that is required for cell viability and Tat transactivation. Moreover, it is suggested built-in intracellular mechanisms allow cells to cope with changes in P-TEFb protein levels [53].

Recently it has been reported that minocycline (MC), a second-generation non-toxic tetracycline, possesses the ability to inhibit NF-κB transcriptional activation and thereby viral replication in microglia [54]. These resident brain macrophages play a central role in AIDS dementia, as they are the primary targets of productive infection in the brain. Besides interrupting LTR activation, this agent also seems to influence the production of cytokines, chemokines, and other substances implicated in AIDS dementia. It appears that MC may act by increasing NF-κB complex formation, resulting in inactive homodimers. This suggests that MC possess the ability to suppress both viral production and inflammation.

### HIV-1 mRNA splicing and nuclear export

**Figure 2:**
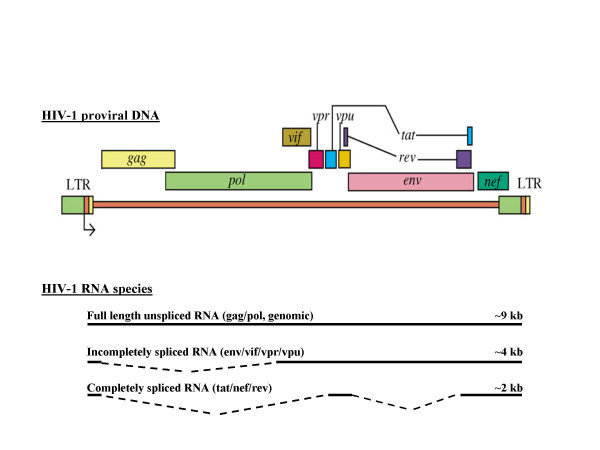
Schematic representation of the HIV-1 provirus and the different RNA species. Gag; group specific antigen, Gag-Pol; group specific antigen-polymerase, Env; envelope, Tat; trans-activator of transcription, Rev; regulator of expression of virion proteins, Nef; negative effector, Vif; virion infectivity factor, Vpr; viral protein r, Vpu; viral protein u, LTR; long terminal repeat.

Viral gene expression can be divided into an early and late phase, which is Rev-independent and Rev-dependent, respectively. In the early phase the newly transcribed mRNA is spliced by the cellular splicing machinery into multiply spliced transcripts, which mainly produces the Tat, Rev and Nef proteins. When Rev has accumulated to a critical level the mRNA production shifts from multiply spliced to the singly spliced and unspliced transcripts, characteristic of the late phase of gene expression. Rev contains an RNA binding motif that directly interacts with stem-loop IIB located within an RNA multi-stem-loop secondary structure, termed the rev response element (RRE), which is present in the env gene of all incompletely spliced viral mRNAs. The RRE can accommodate the binding of at least 8 Rev molecules, and at a certain threshold concentration of Rev protein in the nucleus, functional Rev/RRE complexes are formed, which greatly stimulate the export of unspliced and singly spliced RNA to the cytoplasm where translation can proceed. Nuclear export is mediated by cellular co-factors termed exportin 1 and Ran-GTP, which interacts cooperatively with the Rev nuclear export signal (NES) sequence [55]. The nuclear import of Rev is mediated by the cellular co-factor importin-beta that interacts directly with a NLS sequence in Rev. The host proteins B23 and p32 also interacts with the NLS region and may be involved in nucleolar localization [4,5,56-58]. The fundamental and essential function of Rev has made it a popular target for therapeutic development [57]. Fig. 2 illustrates the structure of proviral DNA and the different RNA species.

Many of the used strategies are based on inhibiting the Rev/RRE interaction. Examples include Rev TNPs, RRE RNA decoys, anti-sense Rev/RRE RNAs, siRNAs, sFvs, nucleases, ribozymes, aptamers, chimeric proteins, and small inhibiting molecules.

The most well characterized Rev TNP is the RevM10 protein, which contains amino acid substitutions within the NES region. The ability of RevM10 to form multimeric structures and to interact with RRE is not hindered by these mutations, but the interaction with cellular co-factors is prevented leading to inhibition of Rev function [7,18,25,57-59]. Making deletions in the NLS sequence generates another kind of Rev TNP, the Rev38. The result is a mutant that accumulates in the cytoplasm where it sequesters the wildtype Rev protein by formation of inactive oligomers, thereby hindering the transport of Rev into the nucleus [18,57]. Likewise, Rev mutants lacking the ability to form multimeric structures (RevSLT26 and RevSLT40) are effective inhibitors. Of the mentioned TNPs, RevM10 is the most potent inhibitor that has been used in clinical trials [57].

RRE RNA decoys act by recruiting the Rev molecules and thereby hindering their interaction with the viral transcript. Potent inhibition has been achieved by overexpression of a 45 nucleotide chimeric tRNA-RRE transcript [7]. This type of decoy unfortunately also binds essential cellular co-factors, which has led to the design of minimal RNA decoys that only contains the Rev binding site. An example is a 41 nucleotide RRE SLIIAB RNA decoy that has been used in clinical trials [6].

sFvs targeted against Rev potently recruit Rev proteins in the cytoplasm, which accelerates the degradation of Rev [7,18,25,57]. In a comparative study the effect of monoclonal sFv targeting either the NES region or the C-terminal region of Rev was compared. The NES-specific sFv demonstrated the best antiviral effect, even though the binding affinity of the C-terminal sFv for Rev was significantly higher [57].

Additional strategies for inhibition of Rev function include, 1) RNA aptamers, possessing a higher affinity for Rev than the RRE sequence, and which recognize other epitopes than the natural RNA binding site on Rev [57], 2) peptides that recognize the NES domain [57], 3) a dominant negative mutant form of Sam68 (Src-associated protein in mitosis), whose natural function is to interact with RRE and thereby partially substitute or synergize with Rev in RRE-mediated gene expression. In particular, a C-terminally deleted mutant of Sam68 inhibits not only Sam68 transactivation of RRE, but also Rev function [60]. Unlike RevM10, which competes with wildtype Rev for binding to RRE in the nucleus, this Sam68 mutant is mainly cytoplasmic, and binds RRE very poorly. However, it retains the ability to bind Rev and the mechanism may involve trapping of Rev in the cytoplasm by direct protein-protein interaction. It is thought that the complex formation of Sam68 and Rev has a masking effect that inactivates the Rev NLS domain. Since the Sam68 NLS domain is deleted in the mutant it will not be able to substitute for the missing Rev NLS domain, thus the HIV-1 replication is inhibited. 4) chimeric proteins. One example is a construct where the Rev protein is covalently attached to a mutant form of the NS1 protein from the Influenza A virus (NS1RM-Rev). It is thought that the fusion protein and wildtype Rev form mixed oligomers, and due to the nuclear retention activity of NS1, which is dominant over the Rev-mediated nuclear export, it results in inhibition of nuclear export of viral transcripts. NS1RM-Rev has an antiviral effect equal to that of the RevM10 mutant [7,57].

As with inhibition of the Tat/TAR interaction, the anti-sense and ribozyme strategies can also be targeted towards the Rev/RRE interaction. Anti-sense directed against RRE will inhibit Rev binding, whereas anti-sense directed against the Rev encoding region will hinder the expression of Rev protein [6]. The anti-sense RNAs can be either unmodified or modified RNAs. E.g. a synthetic phosphorothioate oligodeoxynucleotide targeting Rev mRNA has antiviral activity in chronically infected cells [25]. Unfortunately, this type of anti-sense RNA strategy has shown limited success as a therapeutic agent because of unsolved problems such as efficacy, cell permeability, delivery and cost. Ribozymes targeting RRE or the Rev encoding region hinder viral replication by cleaving the targeted RNA [6,7].

By fusing the Rev protein with a nuclease it is possible to create a nuclease with affinity for the RRE and which therefore has the potential ability to specifically cleave all HIV-1 RNAs carrying the RRE sequence [5]. Also, siRNAs directed against the rev transcript potently inhibit virus replication [49,61].

Finally, strategies based on small molecules that either interact with host cellular proteins or bind to RRE, have also been applied to inhibit Rev function. Due to the ability to interact with cellular proteins, these molecules are often cytotoxic and therefore not usable for eventual clinical trials. Nevertheless they are included here because less toxic derivatives could be developed in the future. An example is Leptomycin (LMB), an antibiotic agent that interacts with the cellular protein CRM1, blocking the binding to the Rev NES domain. A clear disadvantage of LMB is, besides long-term toxicity, its ability to block the transport of other proteins with a NES domain [25,56,57]. An overexpressed truncated version of the nucleopurine, Nup214/CAN, delta-CAN, has shown a closely related mechanism of inhibition. Delta-CAN retains the skill to interact with CRM1 and inhibits Rev function in the same way [56].

Aminoglycoside antibiotics, such as neomycin B and derivatives have shown antiviral effect by binding to RRE and thereby hindering the Rev/RRE interaction. The binding of aminoglycoside antibiotics to RNA is very unspecific, and together with a low selectivity, this drug is unfortunately highly toxic for humans [25,57].

The intercalating dye, pyronin Y, completely blocks the formation of the Rev/RRE complex in vitro. But since pyronin Y also intercalates in DNA, the result is an elevated level of cell death. Other intercalating agents, derivatives of diphenylfuran, have been reported to inhibit the Rev/RRE interaction by causing a conformational change in RRE. These compounds possess the same disadvantages as pyronin Y [25].

### HIV-1 translation

As illustrated in fig. 2 translation of the non-spliced RNA and single-spliced RNA result in the Gag and Gag-Pol proteins, and in the Vpu, Vif, Vpr and Env proteins, respectively. Translation of the completely spliced RNA results in the Tat, Rev and Nef proteins [4,6-8].

Designed anti-sense RNAs directed against sequences located in the 5' UTR of all HIV-1 mRNAs, would be able to hinder the ribosome-complex in completing the translation process and thereby inhibit the protein synthesis [6]. Hairpin ribozymes directed against the U5 region have shown similar antiviral responses [6,7].

A study involving nef shRNAs corresponding to nef-derived miRNAs, which recently have been demonstrated to be produced in HIV-1-infected cells [62], shows the potential to efficiently block RNA stability or mRNA translation. This may indicate that HIV-1 possess the ability to regulate its own replication. Interestingly, another study has shown that HIV-1 putatively encodes five candidate pre-miRNAs, which potentially could target a large number of cellular transcripts indicating that HIV-1 moreover may have the potential to regulate the cellular milieu [63].

### Viral assembly, release and maturation

The virus particle is assembled at the plasma membrane. In this process the Gag and Gag-Pol polyproteins interact with each other by protein-protein interaction, most probably via the capsid (CA) protein domain [64]. The viral genome is packaged in a process in which the packaging signal, Psi, is recognized by the nucleocapsid (NC) protein domain of the Gag protein [65]. Another important function of the NC domain is to mediate the formation of the RNA dimer via a palindromic sequence in the dimer linkage structure (DLS) sequence, which is located in the Psi sequence [4,6]. In addition several cellular tRNAs are packaged. The assembly of the virus particle is partly regulated by the Vpu and Vif proteins. The primary function of the Vpu protein is to mediate the release of the virus particle from the cell surface, by selectively targeting the CD4 protein to a degradation pathway in the endoplasmic reticulum (ER). This permits the release of Env protein from the ER, which may otherwise be complexed with the CD4 protein, and further processing of the Env protein can then proceed [4,5]. A current thought is that Vif, besides influencing the late stages of virion assembly, may also block premature processing of Gag precursor protein by the HIV protease (Pro). This kind of temporal control of Gag processing ensures the availability of the CA, MA and NC peptides, when the assembly of the viral components takes place at the plasma membrane [4,66,67]. In addition, several studies have recently suggested that Vif may possess another important function in which it acts by overcoming the antiviral activity of a cellular cytidine deaminase, APOBEC3G (CEM15) that induces hypermutations in newly synthesized HIV-1 DNA [68,69]. The mechanism by which Vif inhibits APOBEC3G function is unclear, but it is thought to form a complex with APOBEC3G, thus preventing its viral encapsidation. Furthermore, Vif can target APOBEC3G for degradation via the ubiquitin-proteasome pathway.

After virus budding from the cell surface, maturation of the virus particle proceeds. At this stage in the virion life cycle the Gag and Gag-Pol polyprotein are proteolytically cleaved by the protease domain of Gag-Pol precursors. Cleavage of Gag results in the MA, CA, NC and CEL viral proteins. The MA proteins forms a matrix under the viral envelope, the CA proteins condenses to form a conical core surrounding the NC-coated RNA genome and the CEL protein is thought to associate with the Env protein and, in addition to mediating packaging of Vpr.

Cleavage of the Gag-Pol polyproteins, which is made by ribosomal frameshifting during translation of unspliced mRNA, results in the enzymatic proteins IN, RT and Pro. Both the Gag and Gag-Pol precursors associate with the Env protein by protein-protein interactions between the MA domain and the TM protein. After maturation the virus is ready for another round of infection [4]. Strategies directed against this final step in the viral life cycle include RNA decoys, TNPs, chimeric proteins, anti-infectious cellular proteins, sFvs, nucleases, anti-sense RNAs, ribozymes and peptides.

#### Viral assembly

The packaging signal Psi is a highly conserved RNA sequence and is therefore an obvious target for inhibition strategies. The Psi region contains four stem-loops located near the 5' major splice donor and the start of the gag open reading frame and is essential and sufficient for RNA packaging [45].

RNA containing a 1.43 kb region anti-sense to the Psi-gag region has been reported to inhibit packaging of genomic RNA and thus HIV-1 replication with a higher efficiency than the RevM10 mutant [6,7,45]. An antiviral effect has also been observed by targeting anti-sense RNAs against the Psi sequence, which act by hindering the NC domain of the Gag protein in recognizing the packaging signal. Likewise, ribozymes directed against the packaging signal have also been shown to have a significant effect on viral replication [7,6].

Since the Psi element is recognized by the NC domain, one strategy is to design a NC-nuclease fusion protein, which will recognize and cleave all unspliced HIV-1 RNAs. The cleavage process occurs in the cytoplasm and this will also inhibit the production of the Gag and Gag-Pol fusion proteins [5]. Expression of the gag-gene has also been reduced by using anti-sense RNAs complementary to the 5'-leader-gag region or by using ribozymes directed against the gag transcripts [7,18].

The ability of the NC domains to recognize the genomic RNA can be blocked with RNA decoys containing the Psi sequence. These decoys may form dimers with HIV-1 RNA and thereby compete with HIV-1 RNA dimers for packaging into new virus particles [6,45].

The structural proteins, Gag and Env, form multimeric complexes during viral assembly. By means of different kinds of Gag TNPs it has been possible to inhibit this step. Besides inhibiting viral assembly, these Gag TNPs also interfere with the viral release process, the uncoating of the viral genome and the reverse transcription [18]. A limitation of using Gag TNPs is that the gag-gene contains an inhibiting sequence, the CRS, which hinders the expression of the gag-gene if Rev is missing [4,6,18]. The potential effect of Env TNPs has also been tested, but this strategy shows relatively low antiviral activity when compared to Gag TNPs [18].

Given that one function of the Vif protein is to block early processing of the Gag protein, it has inspired the development of a Vif TNP that is missing the blocking function. Applying this protein means that the virus assembly is impaired, but a clear disadvantage is that HIV-1 has the potential to evolve escape mutants [66]. Similar inhibition has been observed by directing anti-sense RNAs against the Vif encoding region [70]. Another strategy relies on the INI1 protein that is the only known host protein directly interacting with HIV-1 integrase. A minimal integrase-binding fragment of INI1, S6, comprising amino acids 183–294, potently inhibits HIV-1 assembly, particle production and replication in a transdominant manner. This inhibiting effect results from direct interaction of the S6 protein with the integrase domain within the Gag-Pol polyprotein. When the S6 protein binds to integrase it is thought to interfere with proper multimerization of Gag and Gag-Pol by steric hindrance. In addition it affects maturation, blocks an interaction of the cellular assembly machinery with Gag-Pol and mediates the mislocalization of viral proteins into different sub-cytoplasmic compartments of the cell. Besides being non-toxic, another favorable feature of S6 is that it is unlikely to be immunogenic, because it is a truncated form of a host protein. In addition, virions with mutations in the S6 binding site will also be defective for interaction with INI1. This minimizes the risk for the development of HIV-1 escape mutants [39].

Recently, a specific inhibition of virus budding was demonstrated by overexpression of an amino-terminal fragment of tumour susceptibility gene 101 (Tsg101) [71]. The role of Tsg101 is to participate in the endocytic trafficking pathway. It is presumed to bind to the Gag polyprotein and subsequently mediate the transport into multivesicular bodies (MVBs), which then carry their cargo towards the cell surface.

By targeting an intracellular sFv specifically against the CD4 binding region of the SU protein, it has been possible to make cells temporarily resistant to HIV-1 infection. This sFv, named sFv105, acts by binding to the Env protein, and traps Env in the ER. This prevents the maturation process in which Env is cleaved into the SU-TM proteins. As a result, Env is prevented from reaching the cell surface [7,18]. Another way to inhibit the maturation of Env is by inhibiting the cellular protease furin [72]. Furin is a member of the mammalian subtilisin-related proprotein convertases that mediate cleavage of the Env protein at a conserved Arg-Glu-Lys-Arg sequence. Peptides that mimic this sequence have been reported to block furin activity. Especially, a polyarginine peptide has shown promising results without showing any toxic side effects on cultured cells *ex vivo *or in mice *in vivo*.

Furthermore, anti-sense RNA approaches directed against the Env message also belong to the applied strategies [70].

Multitarget ribozymes that target different sites in the SU sequence also exert antiviral effects. Some of these designed ribozymes bind and cleave up to nine different conserved regions in the SU sequence [7].

A Gag-nuclease fusion protein can be packed into new virions and thereafter, in the proceeding rounds of infection, efficiently cleave the viral genomic RNA. Since the Gag protein has many essential functions it is unlikely that HIV-1 will develop escape mutants when using the Gag-nuclease. Vpr- and Nef-nuclease fusion proteins also seem to cleave viral RNAs, either during or after viral assembly [5,7].

#### Viral release

A Nef TNP has shown an antiviral effect by inhibiting down-regulation of e.g. the CD4 cell surface protein. It is thought that CD4 may interact with the Env protein present on new viral particles, thus hampering viral release from the cell surface [66]. Likewise, anti-sense RNAs and ribozymes directed against a conserved 14 nucleotide region in the nef-gene also possess an antiviral effect [7].

By overexpressing different kinds of CD4 variants in the infected cell, it is possible to inhibit virus budding. The reason is most likely that the CD4 variants possess the ability to trap and restrain new virions in the cell [19].

#### Viral maturation

The HIV-1 protease plays an important role in virus maturation. As mentioned earlier, the HIV-1 protease cleaves the Gag and Gag-Pol polyproteins to form the structural and enzymatic proteins. Consequently, the protease is a potent target for inhibiting strategies. The current strategies involve protease inhibitors that bind to the active site of the HIV-1 protease and thereby inhibit processing of the Gag and Gag-Pol polyprotein precursors. This results in immature and noninfectious viral particles. The HIV-1 protease is an aspartyl protease and inhibitors have been designed that optimally bind to the catalytic aspartate residues and additionally to the water molecule that is critical for enzymatic action. The inhibitors are transition state analogues that bind the enzyme much more tightly than the natural substrate, making them competitive enzyme inhibitors. Examples of approved protease inhibitors include Saquinavir, Indinavir, Ritonavir, Nelfinavir and Amprenavir [8,11,73].

Other strategies that target Pro involve the application of Pro TNP [7], and the overexpression of chimeric Vpr protein in which the C-terminal region is fused to several cleavage sites recognized by the protease. This will overwhelm the protease activity by a competitive mechanism and impair protease function [7,74]. Finally, anti-sense RNAs targeting the Pol coding region inhibit this last step in the viral life cycle [70].

### Combination of antiviral strategies

By combining the different antiviral strategies, the effectiveness can be increased and the chances of generating escape mutants will be minimized. Examples of combination therapies include ribozymes combined with decoy or anti-sense RNAs [6,75], and decoy RNAs combined with anti-sense RNAs [6,76]. An autoregulated dual-function anti-Tat gene is an example of the latter strategy, in which both a polymeric TAR and an anti-sense Tat are combined in one expression unit [76]. To accomplish gene expression in HIV-1 infected cells, a double-copy retroviral vector, in which gene expression is driven by the HIV-1 LTR, is used. By this approach anti-Tat gene expression is upregulated only in HIV-1 infected cells.

The RT and Pro inhibitors are preferentially not used alone to avoid the risk of generating viral escape mutants. By combining the different kinds of inhibitors (usually a combination of two nucleoside analogues with either a protease inhibitor or a non-nucleoside analogue) significant inhibition of HIV-1 is achieved. This combination strategy is also known as HAART (highly active antiretroviral therapy). Because nucleoside and non-nucleoside analogues act on two different positions on the RT enzyme they will not compete for binding and when used in combination they exhibit a more potent effect. The disadvantage concerning this strategy is the relative strong toxic effects related to these RT drugs. Another problem arises if the prescribed treatment is not exactly followed and resistant viral mutants emerge [31].

When combining strategies involving ribozymes and RNA decoys one can obtain better results than by using one of the strategies. This is clearly illustrated by the tRNA^Val^-RRE-SLII-U5 hairpin ribozyme, in which SLII (stem loop II) contains the Rev protein-binding site that acts as a decoy [6].

In an attempt to interfere with two essential HIV-1 activities at the same time, a double transdominant negative Tat-Rev fusion protein, Trev, has been designed. This fusion protein inhibits both Tat transactivation and Rev mediated nuclear transport [18]. A similar designed fusion protein, Tev, contains the RNA binding domains of both Tat and Rev, and can thus target both TAR and RRE within the HIV-1 RNA. Furthermore, a nuclease was fused to the Tev protein. The result is an inhibition of both early and late viral gene products. Tev contains a NLS and is therefore predicted to act primarily within the nucleus [5].

The combination of an anti-Rev sFv, which targets the Rev activation domain, and a ribozyme that targets RRE, or an RRE RNA decoy, which recruits the Rev molecules, has also shown good results [7]. Promising results have also been described by using a vector expressing three products, the U5 ribozyme, a ribozyme targeted against the Env/Rev encoding regions, and a RRE decoy respectively [18].

### Strategies based upon suicide genes

Conditional expression of suicide genes in cells infected with HIV-1, e.g. by expression from a Tat dependent promoter or under Rev dependent control, has been designed in different versions. Examples of suicide gene approaches include engineering cells with a diphtheria toxin A-chain (DT-A) gene, a cytosine deaminase gene, a herpes simplex virus (HSV) thymidine kinase (tk) gene, and a herpes simplex shutoff (vhs) gene.

DT-A is a very effective cellular toxin that kills cells by blocking the protein synthesis via the ADP-ribosylating elongation factor 2 [77]. Cytosine deaminase mediates cell death through the conversion of 5-fluorocytosine to the potent cytotoxic agent 5-fluorouracil [18], and the HSV thymidine kinase mediates cell death by metabolizing nucleoside analogues, such as Ganciclovir and Acyclovir, into toxic analogues [7,18]. The latter strategy has been further explored in a study involving a live-attenuated form of HIV-1 in which the nef gene has been deleted and instead engineered to express the thymidine kinase gene [78]. This marked live-attenuated virus vector may be useful to accrue baseline information on the immunological benefits of a replicating vaccine. The safety profile of such a vaccine vector is supported by the possibility to remove cells harboring integrated proviral genomes if necessary.

Another approach involves the design of a hybrid molecule consisting of the human CD4 and a modified version of the Pseudomonas exotoxin A (CD4-PE40). This molecule binds to infected cells by a CD4-SU interaction at the cell surface and, after uptake, the exotoxin inhibits protein synthesis, thus leading to cell death [7].

Finally, the last example involves a modified apoptosis-promoting caspase-3 protein, Tat-Casp3, which acts by cleavage of the inhibitor of caspase-activated DNase, resulting in the activation of caspase-activated DNase and, ultimately, cell death. In this design the endogenous cleavage sites have been substituted with the HIV proteolytic cleavage sites, and the Pro domain of the modified Casp3 protein was removed and substituted with the Tat transduction domain. Hence, the fusion protein is only activated by the HIV protease in infected cells, resulting in apoptosis, whereas in uninfected cells it remains in the inactive zymogen form. Tat-Casp3 proteins may also be packaged inside the virion and kill the virion after it buds from the cell and/or initiate apoptosis immediately after subsequent infection of a cell [79].

## Conclusion

In spite of the astonishing diversity of methods developed as antivirals against HIV-1, still many problems remain. Perhaps the most difficult problem to solve is the remarkable ability of HIV-1 to evade the different inhibiting strategies. The selection pressure enforced by the treatment may result in the selection of escape mutants that are more pathogenic than the original virus. For instance: Blocking the interaction with CCR5, which is the primarily used co-receptor, could result in usage of both the CCR5 and the CXCR4 co-receptors or CXCR4 alone. The outcome will be an accelerated reduction of CD4^+ ^T-cells and thereby a progression of the disease [80]. The risk of evolution of a virus variant that uses new co-receptors is not unthinkable. To obtain long-term inhibition and to avoid escape mutants, it is necessary to combine the different strategies, so that several steps in the viral life cycle are inhibited at the same time.

RNAi is a very promising novel approach that in principle will provide a large number of new targets that may be combined, but unfortunately one of the biggest hurdles is the in vivo delivery problem, which needs to be addressed. A gene therapy approach may be used to make hematopoietic stem cells resistant to HIV-1, which could eventually lead to (partial) restoration of the immune system.

In spite of the advanced technology used in the different virus intervention strategies and the rapidly growing knowledge about the molecular biology of HIV-1, it has not yet been accomplished to block HIV-1 replication completely. Hopefully, scientists will succeed to push the balance of the virus-host battle in the right direction so that the immune system will be able to handle the remaining viruses, or vaccine strategies may be added to clear the virus. Independently of the path taken, it will most likely require that the latent reservoirs of virus are activated to make them more vulnerable to treatment. A combination of these directions may eventually lead to a complete eradication of the virus in infected patients.

## List of abbreviations used

Aids; acquired immunodeficiency syndrome

HIV-1; human immunodeficiency virus type 1

shRNA; short hairpin RNA

siRNA; small interfering RNA

RNAi; RNA interference

miRNA; microRNA

dsRNA; double stranded RNA

Gag; group specific antigen

CA; capsid protein

MA; matrix protein

NC; nucleocapsid protein

CEL; core-envelope-link protein

Gag-Pol; group specific antigen-polymerase

RT; reverse transcriptase

IN; integrase

Pro; protease

Env; envelope

SU; surface protein

TM; transmembrane protein

Tat; trans-activator of transcription

Rev; regulator of expression of virion proteins

Nef; negative effector

Vif; virion infectivity factor

Vpr; viral protein r

Vpu; viral protein u

TAR; trans-activation responsive element

RRE; rev response element

Psi; packaging signal

DLS; dimer linkage structure

PBS; primer binding site

LTR; long terminal repeat

R; repeat

U5; unique 5'

U3; unique 3'

ER; endoplasmatic reticulum

NES; nuclear export signal

NLS; nuclear localization signal

TNP; transdominant negative protein

sFv; intracellular single-chain antibody

Mab; monoclonal antibody

TRTI; template analogue RT inhibitor

NRTI; nucleoside reverse transcriptase inhibitor

NNRTI; non-nucleoside reverse transcriptase inhibitors

HAART; highly active antiretroviral therapy

SELEX; systematic evolution of ligands by exponential enrichment

DCQA; dicaffeoylquinic acid

LMB; leptomycin

Tsg101; tumour susceptibility gene 101

MVB; multivesicular body

DT-A; diphtheria toxin A-chain

tk; thymidine kinase

RANTES; regulated upon activation, normal T-cell expressed and secreted beta-chemokine

PBMC; human peripheral blood mononuclear cell

CV-N; Cyanovirin

CyPA; human cyclophilin A

## Competing interests

The author(s) declare that they have no competing interests.
